# Dietary Polysaccharides and the Regulation of Blood Glucose and Lipid Parameters—A Narrative Review

**DOI:** 10.3390/nu18132143

**Published:** 2026-07-02

**Authors:** Omorogieva Ojo, Yemi Onilude, Osarhumwese Osaretin Ojo, Victoria Apau, Ivy Kazangarare, David Agyapong, Joanne Brooke, Xiaohua Wang

**Affiliations:** 1 School of Health Sciences, Faculty of Education, Health and Human Sciences, University of Greenwich, Avery Hill Campus, London SE9 2UG, UK; o.onilude@greenwich.ac.uk (Y.O.); v.apau@greenwich.ac.uk (V.A.); ivy.kazangarare@greenwich.ac.uk (I.K.); d.agyapong@greenwich.ac.uk (D.A.); 2 South London and Maudsley NHS Foundation Trust, London SE13 6LH, UK; osarhumwese.ojo@nhs.net; 3 Centre of Social Care, Health, and Related Research, Birmingham City University, Westbourne Rd., Birmingham B15 3TN, UK; joanne.brooke@bcu.ac.uk; 4 The School of Nursing, Soochow University, Suzhou 215006, China; wangxiaohua@suda.edu.cn

**Keywords:** dietary polysaccharides, blood glucose, type 2 diabetes, lipids, satiety, gut microbiota

## Abstract

The increase in the prevalence of non-communicable diseases globally has been attributed in part to poor lifestyle choices, including unhealthy dietary habits. Dietary polysaccharides, including resistant starch and non-starch polysaccharides, have gained increasing attention due to their potential role in the regulation of glucose and lipid metabolism. Therefore, the aim of this review was to evaluate the role of dietary polysaccharides in the regulation of blood glucose and lipid parameters. Method: A narrative review approach was adopted for this review. Searches were conducted through EBSCOHost and involved the following databases: Medline, APA PsycInfo, CINAHL Plus with Full Text, Psychology and Behavioural Sciences collection, Academic Search Premier and APA PsycArticles. Searches were conducted on 14 April 2026 and covered all records available from database inception to the search date. Search terms were combined using Boolean operators (AND/OR). The reference list of articles was also searched for more articles. Results: Twenty-one studies from thirteen different countries were included in this review. Based on narrative synthesis, five themes were identified: the effects of dietary polysaccharides on glycaemia, insulin, lipids, energy intake and satiety/appetite. The findings demonstrated considerable heterogeneity across studies. While several studies reported improvements in fasting glucose, postprandial glucose, glycated haemoglobin and insulin responses following resistant starch and non-starch polysaccharide interventions, other studies found no significant effects on glycaemic control or insulin levels. Lipid outcomes were similarly inconsistent, although some studies reported reductions in total cholesterol and low density lipoprotein cholesterol. Effects on energy intake and satiety varied according to the type and physicochemical characteristics of the polysaccharide investigated. Conclusion: The findings of this review suggest that dietary polysaccharides may contribute to improvements in glucose control and lipid metabolism, although the magnitude and consistency of these effects vary across populations, intervention types and study designs. The most frequently reported beneficial findings related to blood glucose parameters, although substantial heterogeneity remained across studies. Further, well-designed studies, including randomised controlled trials with longer durations, are needed to fully establish the role of dietary polysaccharides in the control of blood glucose and lipid parameters.

## 1. Introduction

Metabolic diseases including obesity, insulin resistance and type 2 diabetes continue to increase globally and are associated with poor dietary habits, sedentary lifestyle and gut microbiota dysbiosis [1,2,3]. Consequently, there is growing interest in dietary approaches that may improve glucose homeostasis and lipid metabolism.

Dietary polysaccharides are natural macromolecules derived from the condensation of different monosaccharide molecules and have ten or more monomeric units that are linked together by glycosidic bonds [4,5]. Dietary polysaccharides are common components of the human diet and can be found in various living organisms including plants, animals, fungi and marine algae, as well as in fruits, vegetables and herbs consumed daily [1,6]. Polysaccharides are the most abundant types of carbohydrates [6]. Furthermore, plant polysaccharides are among the most active bioactive compounds in plants, which has highlighted their significance in human nutrition, health and biological activities, including the regulation of glucose and lipid metabolism [4,7]. Given the increasing prevalence of metabolic diseases, dietary polysaccharides have attracted considerable attention because their structural and physicochemical properties may influence digestion, nutrient absorption, postprandial metabolic responses and gut microbial activity [6,8]. However, dietary polysaccharides comprise a diverse group of compounds that differ substantially in their structure, solubility, viscosity and fermentability that may contribute to the variation in their metabolic effects [7,8]. Understanding these differences is important when evaluating their potential role in glucose and lipid regulation.

### 1.1. Sources and Classification of Dietary Polysaccharides

The categorisation of bioactive polysaccharides is based on broad categories including, origin, application, solubility, structure, and chemical composition [5]. For example, animal sources include heparin, plant-derived polysaccharides include pectin, and exopolysaccharides are bacterial in origin [5]. Polysaccharides may be further classified based on the composition of the monosaccharide units, and these classifications include, homopolysaccharides or homoglycans (e.g., starch, cellulose and glycogen), which comprise only one type of monosaccharide, while heteropolysaccharides or heteroglycans (e.g., pectin, heparin), are made up of two or more different monomeric units [5,6]. The classification of polysaccharides is clinically important because their structural characteristics may influence digestibility, viscosity, fermentability and metabolic effects, including glycaemic regulation and lipid metabolism.

In relation to the biological functions, polysaccharides can be divided into two categories; the storage polysaccharides such as starch and the structural polysaccharides including cellulose and pectin [7]. The storage polysaccharides are found primarily in the cytoplasm, while the structural polysaccharides exist mainly in the primary and secondary cell walls [6]. The structural/cell wall polysaccharides or non-starch polysaccharides are derived primarily from plants, fungi and algae [8]. Both storage and structural polysaccharides contribute to complex physiological processes, are useful sources of fibres in human and animal diets and influence physical function and health [6,9].

While some dietary polysaccharides, such as starch, are digestible and can be absorbed by the body to provide energy, others such as fibre cannot be digested and absorbed by the body due to the lack of enzymes in the body [1,9]. The latter dietary polysaccharides can be classified as either fermentable or non-fermentable polysaccharides [1].

### 1.2. Starches

Starch is stored mainly in plant seeds, such as legumes, mangoes, wheat, maize and rice, or roots, such as potatoes, in granular form and consists of a pure glucose polymer [10,11]. Starch is made up of linear amylose and highly branched amylopectin molecules [11]. Based on in-vitro amylolysis and rates of digestion, starches can be broadly classified into three types; rapidly digestible starch, slowly digestible starch, and resistant starch (RS) [11]. The differences in starch types are based on the time they take to digest in the small intestine [11]. While rapidly digestible starch is rapidly converted to glucose through enzymatic digestion within 20 min; slowly digestible starch takes more than 20 min to convert to glucose through enzymatic digestion, and RS is resistant to hydrolysis by enzymatic digestion [11]. Due to the similarity in the physiological effects on humans, including its non-digestibility and being non-viscous and highly fermentable, RS is often classified as a type of dietary fibre [8,11].

The rate of digestion of starch is often related to the glycaemic index of food [12]. Thus, foods that contain dietary carbohydrates, break down quickly during the process of digestion (e.g., white bread) and are rapidly absorbed into the blood stream are classified as foods with high glycaemic index (GI) [12]. On the other hand, low GI foods such as legumes, lentils, and oats usually contain carbohydrates that slowly break down during digestion and are slowly assimilated [12]. GI has been defined as a measure of the relative rate of digestibility of available carbohydrates of the food compared to a reference food, which is usually glucose [12].

RS can be classified into five types based on its structure and properties [10,11,13]: Type 1 (RS1) is physically inaccessible to digestion and can be found in seeds, cereals and pulses; Type 2 (RS2) is a type of raw granular starch and can be found in grains and potatoes; Type 3 (RS3) is a gelatinized and retrograded starch produced during food processing; Type 4 (RS4) is chemically modified starch; and Type 5 (RS5) is lipid-modified starch, which may be produced during thermal processing in the presence of lipids.

While the compact structure and granular form of amylose and amylopectin limit accessibility of types 1 and 2 RS to digestive enzymes, it is the heat or chemical treatment in types 3, 4, and 5 that enhance their resistance to digestion [10].

### 1.3. Non-Starch Polysaccharides (NSP)

The classes of non-starch polysaccharides (monomeric units ≥ 10) include the following [10]: cellulose and hemicellulose; mannans and heteromannans; pectins; gums and mucilages; inulin; polydextrose; and resistant dextrins.

While native cellulose is found mainly in plant cell walls, its by-products may be useful as functional additives in foods [14]. Cellulose is not soluble in water, and it is not digestible by human enzymes; however, it can be fermented by microbes in the gut to varying degrees [8]. On the other hand, hemicellulose may be seen as that part of cell wall polysaccharides that is not cellulosic and are only soluble in alkaline solutions [8]. Pectin and guar gum are found in plant cell walls and are soluble, viscous, fermentable non-starch polysaccharides [8,14]. Furthermore, guar gum is often classified as a galactomannan, comprising galactose and mannose units [8,14].

### 1.4. Use of Dietary Polysaccharides

Plant polysaccharides are often involved in diverse biological activities, including hypoglycaemic, antioxidant, and immunomodulatory effects, which have a significant benefit for human health [7,15]. Furthermore, important physicochemical properties, such as thickening, emulsifying and stabilising abilities, are useful in the food, pharmaceutical and cosmetic industries [7]. Starch found in cereals, potatoes, cassava, legumes and bananas and other storage carbohydrates are primary sources of energy in all diets, while cell wall polysaccharides are the primary components of dietary fibre [7,8]. Plant polysaccharides are also useful as multifunctional food additives due to their unique physicochemical properties [7].

On the other hand, the dietary polysaccharides, including dietary fibre that cannot be digested or absorbed by the body, are often used as substrates or sources of energy by the gut microbiota for microbial growth through fermentation and the production of short chain fatty acids (SCFAs) [1]. This process leads to the proliferation of beneficial bacteria in the gut [1]. The SCFAs are involved in a range of metabolic activities and are useful in the prevention and management of metabolic diseases, including type 2 diabetes and hyperlipidaemia [1,15]. Although dietary polysaccharides have been widely investigated for their potential metabolic benefits, the available evidence remains diverse and, at times, inconsistent [11]. Studies have reported varying effects on glycaemic control, insulin responses, lipid metabolism, appetite regulation and energy intake [11,16]. These differences may reflect variation in the source and type of polysaccharide investigated, intervention dose and duration, participant characteristics and outcomes assessed [11,16]. Therefore, there remains a need to synthesise the available evidence to provide a clearer overview of the metabolic effects associated with dietary polysaccharides and to identify areas where gaps and uncertainty remain.

### 1.5. Why This Review Is Important

The increasing prevalence of type 2 diabetes and other metabolic diseases, including insulin resistance and obesity, is in part the result of unhealthy diet consumption and increased systemic and tissue inflammation caused by increased systemic levels of bacterial endotoxemia due to the loss of gut microbial eubiosis [1,2,3]. Therefore, the promotion of healthy gut microbiota involving the use of dietary polysaccharides may lead to eubiosis, which could be beneficial in the prevention and management of metabolic diseases [1,17]. The potential role of dietary polysaccharides in metabolic health has attracted increasing research attention because of their possible effects on glycaemic regulation, insulin responses, lipid metabolism, appetite and energy intake [5,18]. Although numerous studies have investigated the metabolic effects of dietary polysaccharides, findings relating to glucose regulation, insulin responses and lipid metabolism have not always been consistent across populations and intervention types [11,16].

Given the breadth and heterogeneity of the literature, a narrative review was considered appropriate for this topic. Narrative reviews are particularly useful for synthesising evidence derived from studies that differ in their design, intervention characteristics, participant populations and outcome measures [19]. Previous reviews focused mainly on the extraction techniques, structural characteristics and biological activities of dietary polysaccharides [5,7].

Therefore, this narrative review provides the opportunity to present a broad perspective of the role of dietary polysaccharides in the control of blood glucose and lipid metabolism in populations with different health and metabolic status [19].

Aim: The aim of this review was to examine the role of dietary polysaccharides in the regulation of blood glucose and lipid parameters.

## 2. Materials and Methods

As this review sought to examine evidence derived from studies with diverse designs, populations, interventions and outcome measures, a narrative review approach was adopted. A narrative review supports the appraisal and synthesis of published research on a topic, through summarising and interpreting all relevant findings from primary studies. This approach supports an overview of the topic, whilst developing new insights and advancing the field of study through applying a different perspective [20]. Therefore, the flexible, rigorous and practice approach of narrative reviews was an appropriate approach for our review [21].

Searches for relevant articles were carried out through EBSCOHost, encompassing a range of databases: Medline, APA PsycInfo, CINAHL Plus with Full Text, Psychology and Behavioural Sciences collection, Academic Search Premier and APA PsycArticles. Searches were conducted on 14 April 2026 and covered all records available from database inception to the search date. The reference lists of the identified articles were also searched for more articles. Search terms were combined using Boolean operators (AND/OR) and included; “Non starch polysaccharides” AND (Glucose control or glucose regulation); “(Starch or resistant starch) AND (Glucose control or glucose regulation or lipid control or fat control); “Dietary polysaccharides” AND “glucose metabolism”; “Dietary polysaccharides” AND “lipid metabolism”.

Two researchers (OO and OOO) conducted the searches, one carrying out the initial searches and the other repeating the searches.

### 2.1. Data Collection

**Figure 1:**
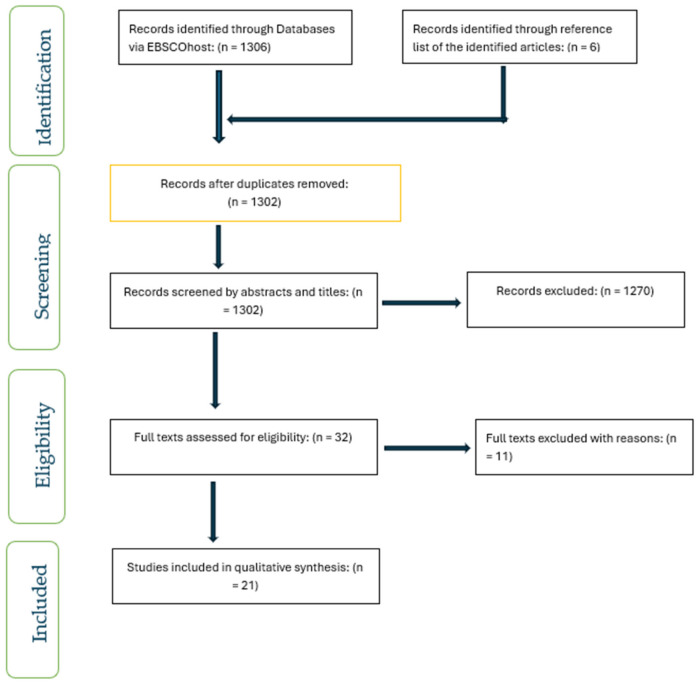
Flow diagram of included studies [22].

The articles were also screened for eligibility by two researchers (OO and OOO), using a flow diagram [Figure 1] [22]. Differences between researchers were resolved through discussion, while duplicates were removed through manual review and verification.

### 2.2. Study Selection

Inclusion criteria: Primary research studies involving human subjects, dietary polysaccharides and blood glucose and/or lipids were included in the review. Furthermore, articles were included if written in English.

Exclusion Criteria: In vitro studies and those involving animals, co-administration of dietary polysaccharides and other products, not written in English, reviews, and with outcomes not related to blood glucose and lipids were excluded from the review. Abstracts and Grey literature were excluded.

### 2.3. Data Extraction and Management

Four researchers (YO, VA, IK and DA) extracted the qualitative data from the studies included in the review and this was crosschecked by a fifth researcher (OO). Differences between researchers were resolved through discussion.

### 2.4. Quality/Risk of Bias Evaluation

Two researchers (YO and VA) conducted the quality assessment using the Cochrane Risk of Bias tool for randomised controlled studies [23] for sixteen studies and the Risk of Bias In Non-randomized Studies—of Interventions [24] for five studies.

Following the identification and selection of relevant studies, key information, including study characteristics, participant demographics, intervention type and reported outcomes, were extracted and summarised.

## 3. Results

Twenty-one studies were included in this review (Table 1 and Supplementary Table S1). Of these studies, three each were conducted in the USA [25,26,27] and the UK [28,29,30], two each were carried out in Canada [31,32], Mexico [33,34], Sweden [35,36] and South Korea [37,38], and one each was conducted in Japan [39], Turkey [40], Spain [41], Taiwan [42], China [43], Thailand [44] and Pakistan [45]. Fourteen studies were randomised cross over studies, while four studies were randomised controlled studies (Table 1). The remaining three studies included an interventional clinical study, an experimental clinical trial and a clinical intervention study.

### 3.1. Quality/Risk of Bias Evaluation of Included Studies

**Figure 2:**
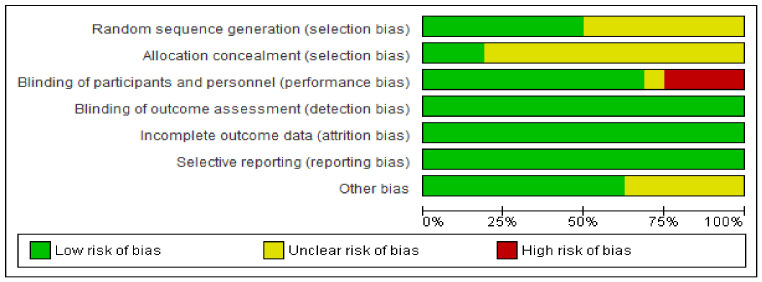
Risk of bias graph of the included studies [25,26,27,28,29,30,31,32,33,34,35,36,37,38,39,40,41,42,43,44,45].

**Figure 3:**
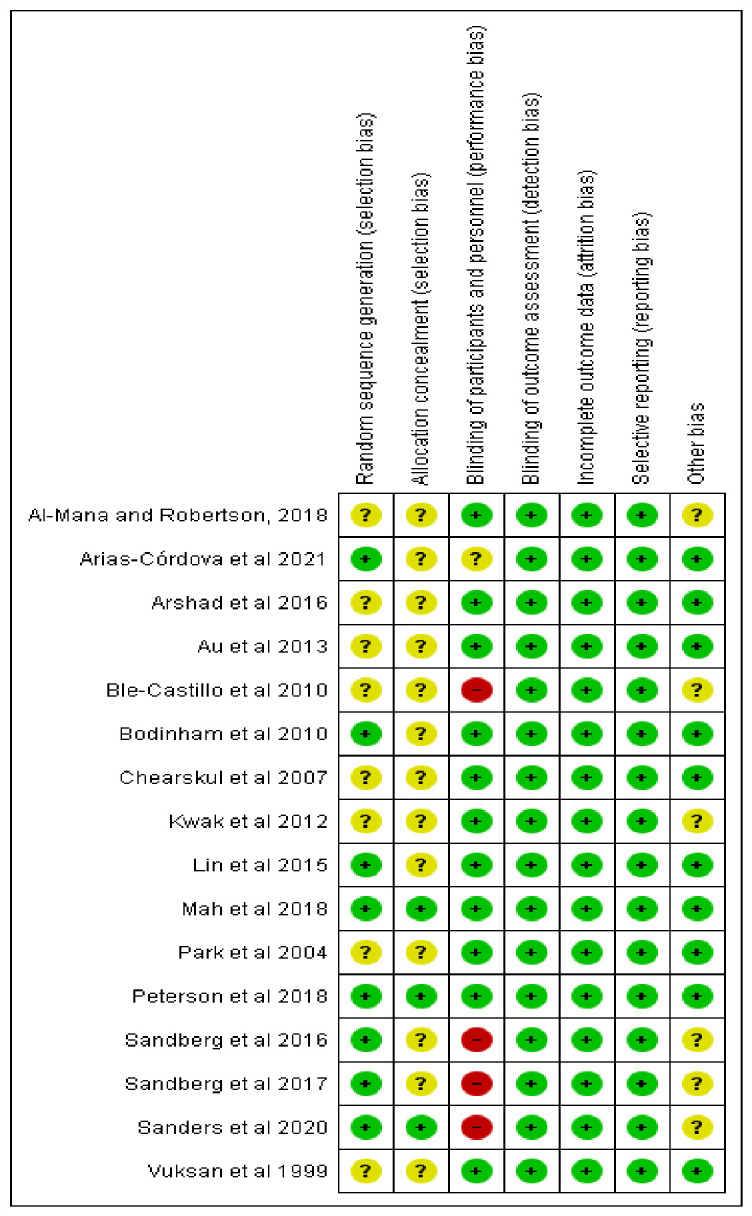
Risk of bias summary of the included studies [25,26,27,28,29,30,31,32,33,34,35,36,37,38,39,40,41,42,43,44,45].

There was low risk of bias in all the studies in relation to blinding of outcome assessment (detection bias), incomplete outcome data (attrition bias) and selective reporting (reporting bias) (Figure 2 and Figure 3). There were unclear risks of bias in some of the studies in respect of random sequence generation [28,30,31,32,34,37,38,45], allocation concealment [28,29,30,31,32,33,34,35,36,37,38,39,40,41,42,43,44,45] and other bias [27,28,34,35,36,37], and four studies [27,34,35,36] showed high risk of bias in relation to performance bias (Figure 2 and Figure 3).

With respect to risk of bias assessment in non-randomized studies, most of the studies showed low or moderate risk of bias in all the domains assessed. However, high risk of bias was observed in Huang et al. [43], Mesa Garcia et al. [41] and Ueno et al. [39] in relation to bias due to confounding factors.

Table 1 demonstrates substantial variation across the included studies in terms of participant populations, intervention characteristics and metabolic outcomes assessed. The studies investigated a range of dietary polysaccharides, including resistant starch, glucomannan and other non-starch polysaccharides, in healthy individuals, overweight or obese participants, and people with impaired glucose regulation or type 2 diabetes (Table 1 and Supplementary Table S1). Although several studies reported beneficial effects on glycaemic control, insulin responses or lipid parameters, the findings were not uniform across all interventions and populations. These differences highlight the heterogeneous nature of the available evidence and support the need for a narrative synthesis of the findings. To facilitate comparison of metabolic outcomes across studies, Table 2 summarises the dietary polysaccharide classifications and their reported effects on glucose, insulin, lipids, energy intake and satiety.

As shown in Table 2, the metabolic effects of dietary polysaccharides varied according to polysaccharide type and outcome assessed. While improvements in glycaemic control were reported in several studies, findings relating to insulin responses, lipid outcomes, energy intake and satiety were more variable. Based on narrative synthesis, the following themes emerged: the effect of dietary polysaccharides on glycaemia, insulin, lipids, energy intake, and feeling of satiety/appetite.

### 3.2. The Effect of Dietary Polysaccharides on Glycaemia

Overall, the evidence suggests that dietary polysaccharides may contribute to improvements in glycaemic control, although the magnitude of effect varied considerably across studies. Despite this, the effect observed in studies involving individuals with overweight/obesity, impaired glucose regulation or type 2 diabetes were comparable to metabolically healthy populations. For example, of the 13 studies involving dietary polysaccharides in people with overweight/obesity, impaired glucose regulation or type 2 diabetes, eight studies either significantly decreased or lowered blood glucose parameters compared to five out of eight studies involving healthy participants.

The role of dietary polysaccharides in glycaemic control has been subdivided into two areas: the effect of resistant starch (RS) on glycaemia and the effect of non-starch polysaccharides on glycaemia.

#### 3.2.1. The Effect of Resistant Starch on Glycaemia

The effect of resistant starch on glycaemia varied across the studies included (Table 1 and Table 2). While some studies showed no significant effect of RS on glycaemia, six of the studies [25,27,37,38,41,42] revealed that RS significantly reduced either fasting glucose, postprandial glucose and/or glycated haemoglobin compared to the control or baseline in the populations studied. For example, Kwak et al. [37] showed that RS significantly decreased postprandial glucose compared to the control in adults with impaired fasting glucose, impaired glucose tolerance, or newly diagnosed T2D. Similarly, Lin et al. [42] also found that RS significantly reduced blood glucose in healthy subjects and in patients with T2D compared to the control, while Mah et al. [25] noted a significant reduction in postprandial glucose compared to the control in healthy adults. Mesa García et al. [41], observed that RS significantly decreased glycated haemoglobin compared to baseline in patients with T2D, while Park et al. [38] found a significant reduction in the mean fasting serum glucose concentrations compared to the control in healthy overweight subjects. Sanders et al. [27] also found significantly lower fasting plasma glucose compared to control in adult participants. In the study by Sandberg et al. [36], consisting of resistant starch 2 compared to the white wheat flour bread reference meal, the evening meal consisting of whole grain rye flour and rye kernels bread (RFB/RKB) + RS decreased responses for glucose.

#### 3.2.2. The Effect of Non-Starch Polysaccharides on Glycaemia

Most of the studies involving NSP [30,39,43,44,45] showed either fasting glucose, postprandial glucose and/or glycated haemoglobin were significantly reduced following treatment compared to control. While Chearskul et al. [44] found long-term use of glucomannan significantly reduced postprandial glucose in people with T2D, Huang et al. [43] noted konjac food rich in glucomannan significantly reduced fasting glucose, postprandial glucose and glycated haemoglobin in people with T2D compared to the control. The study by Onyechi et al. [30] also observed NSP significantly reduced plasma glucose compared to the control in most postprandial time points in healthy subjects. Ueno et al. [39] showed glucomannan significantly reduced glycated haemoglobin and fasting plasma glucose compared to control in patients with T2D. In one study including carrageenan, guar gum, and alginate, guar gum and alginate resulted in significantly lower cumulative blood glucose (0–170 min) compared to the control [45], while in another study, which involved starch and NSP, blood glucose was significantly reduced [35].

### 3.3. Dietary Polysaccharides on Insulin

The evidence relating to insulin responses was less consistent than that observed for glycaemic outcomes. Furthermore, the findings of the studies reporting on the effect of RS and NSP on insulin levels were varied. While several studies reported reductions in postprandial insulin concentrations or improvements in insulin sensitivity following resistant starch interventions, other studies observed little or no effect. Only eight of the 19 studies reporting on insulin found that dietary polysaccharides had significant effect on insulin.

In particular, five studies [29,35,36,37,42] observed RS either significantly lowered postprandial insulin response or significantly reduced fasting serum insulin. On the other hand, Ble-Castillo et al. [34], noted RS increased insulin sensitivity.

The study by Onyechi et al. [30] reported African plant foods rich in NSP significantly reduced insulin at various postprandial time points, while Ueno et al. [39] noted the index for insulin secretion significantly increased following intake of konjac and konjac-based foods.

### 3.4. Dietary Polysaccharides on Lipids

The findings relating to lipid metabolism were heterogeneous. Although several studies reported reductions in low-density lipoprotein (LDL) cholesterol, total cholesterol or free fatty acids following administration of dietary polysaccharides, these effects were not consistently observed across all interventions. Positive lipid outcomes were found in studies involving glucomannan and resistant starch interventions among participants with metabolic abnormalities.

Some of the studies found RS had no effect on total, LDL or high-density lipoprotein (HDL) cholesterol, triglyceride, and free fatty acids in overweight/obese adults with prediabetes [26] or no significant effect on free fatty acids and triglycerides in healthy young adults [35]. However, Park et al. [38] observed RS significantly lowered serum total cholesterol and serum LDL cholesterol compared to the baseline in overweight/obese adults. Similarly, Sanders et al. [27] showed RS significantly lowered postprandial free fatty acid concentrations compared to the control in overweight/obese adults at risk of T2D.

With respect to studies involving glucomannan and patients with T2D, Chearskul et al. [44] observed the mean LDL-cholesterol concentration after receiving glucomannan was significantly less than the placebo, while Vuksan et al. [32], noted that, although there was significantly reduced total:HDL cholesterol ratio compared to the control, the effect on total, LDL, HDL cholesterol and triglyceride was not significant. Furthermore, Huang et al. [43] found no significant effect of glucomannan on lipids except in triglyceride, in patients with hypertriglyceridemia.

### 3.5. Dietary Polysaccharides on Energy Intake

Evidence relating to energy intake was limited and somewhat inconsistent. While some studies demonstrated reductions in subsequent energy consumption following dietary polysaccharide interventions, others reported no significant effect. Al-Mana and Robertson [28], Arias-Córdova et al. [33] and Sanders et al. [27] reported no significant effect of RS on daily energy intake; however, Arshad et al. [45] found caloric intake following administration of alginate and guar gum was significantly lower. Furthermore, Bodinham et al. [29] and Tekin et al. [40] found RS significantly lowered energy intake. With respect to the effect of glucomannan, Chearskul et al. [44] found no significant difference in daily total energy intake between the intervention and control groups.

### 3.6. Dietary Polysaccharides on Feeling of Satiety/Appetite

The effects of dietary polysaccharides on satiety and appetite regulation also varied across studies. Overall, the available evidence suggests a potential role for certain dietary polysaccharides in appetite regulation, although the strength of evidence remains limited. For example, RS was shown to increase satiety compared to white bread [40]. However, Al-Mana and Robertson [28] found no significant differences in qualitative feelings of satiety and the subjective appetite ratings between RS and placebo. Similarly, Bodinham et al. [29] reported RS had no significant effect on subjective appetite ratings.

While the rye kernel significantly increased the subjective feeling of satiety at fasting and during the course of the entire experimental day compared to the control [35], the subjective feeling of hunger and desire to eat were significantly reduced after the rye kernels bread compared to the control [35]. Furthermore, rye flour bread significantly increased the feeling of satiety and decreased feelings of hunger [36].

Although Arshad et al. [45] found the post-treatment average appetite was suppressed by alginate and guar gum, Ueno et al. [39] reported that appetite remained unchanged in two-thirds of participants following the administration of konjac and konjac products. Furthermore, Chearskul et al. [44] noted the appetite scores during treatments with glucomannan and the placebo did not differ.

## 4. Discussion

This narrative review examined evidence on the role of dietary polysaccharides in the regulation of blood glucose and lipid parameters. Overall, the findings suggest that dietary polysaccharides may influence glycaemic control, insulin responses, lipid metabolism, energy intake and satiety, although the direction and magnitude of effects varied considerably across studies. This heterogeneity is an important finding of the review and indicates that the metabolic effects of dietary polysaccharides should not be interpreted as uniform across all populations, intervention types or clinical contexts.

The most consistent findings were observed in relation to glycaemic outcomes. Several studies reported reductions in postprandial glucose, fasting glucose or glycated haemoglobin following interventions involving resistant starch, glucomannan or other non-starch polysaccharides [30,37,38,39,43,44]. However, these effects were not consistently demonstrated across all studies, with some investigations reporting little or no significant improvement in glycaemic outcomes [26,28,31,33].

Variation in the type and physicochemical properties of the polysaccharides investigated may also help explain the inconsistent findings. Resistant starch, glucomannan, guar gum, alginate, rye-based polysaccharides and other non-starch polysaccharides differ in solubility, viscosity, fermentability, molecular structure and resistance to digestion [1,7,8,9,10,11]. These properties may influence gastric emptying, carbohydrate digestion, intestinal absorption and fermentation, which may in turn affect postprandial metabolic responses [1,8,11]. Therefore, it may be inappropriate to treat dietary polysaccharides as a single homogeneous category when interpreting their metabolic effects.

The evidence relating to insulin responses was less consistent than the evidence relating to glycaemic outcomes. Some studies reported reduced postprandial insulin responses or improved markers of insulin sensitivity following resistant starch interventions [25,29,37], whereas others found no significant effect [28,31,38]. This suggests that improvements in blood glucose may not always be accompanied by measurable changes in insulin outcomes, particularly where intervention duration is short or where participants have different baseline insulin sensitivity. It is also possible that variation in the assessment of insulin outcomes across studies limited direct comparison of findings.

Lipid outcomes were also heterogeneous. Some studies reported reductions in LDL cholesterol, total cholesterol or free fatty acids [38,44], while others showed little or no lipid-modifying effect [26,33,34,41]. These inconsistencies may reflect differences in intervention duration, dose, baseline lipid status and the type of polysaccharide used.

With respect to caloric intake and satiety/appetite, some of the studies found RS significantly lowered energy intake and subjective feeling of satiety, although Chearskul et al. [44] found no significant difference in daily total energy intake and appetite scores during treatments with glucomannan between the intervention and control groups. These findings suggest that appetite-related outcomes may depend on the structural properties of the polysaccharide, the food matrix in which it is consumed and whether satiety was measured subjectively or through subsequent food intake.

Although the findings of this review generally support a beneficial role of dietary polysaccharides in metabolic regulation, the evidence was not entirely consistent across studies. Variations in study design, duration of intervention, participant characteristics, baseline metabolic status, source of polysaccharides and dosage administered may partly explain the heterogeneity observed across outcomes. Consequently, the effects of dietary polysaccharides should be interpreted within the context of the specific intervention and population under investigation. According to Shukurjonov et al. [5], the physicochemical characteristics of plant polysaccharides such as solubility, fermentability and viscosity contribute significantly to their metabolic properties. Furthermore, structural and environmental factors such as molecular weight, concentration, polysaccharide type, degree of branching and conditions including temperature, pH and ionic strength have effects on the viscosity of polysaccharides, which is a reflection of their resistance to flow when dissolved in a solvent [5]. These properties may affect nutrient digestion, glucose absorption, gastric emptying, microbial fermentation and short chain fatty acid production, thereby contributing to differences in metabolic outcomes observed between studies.

The findings of the review confirm the results of previous reviews [11,46]. For example, Kim et al. [11] found diets that are rich in RS may be useful in controlling glucose homeostasis. RS has been shown to have functional properties with potential to regulate metabolism and gut microbiota [18]. Furthermore, Weber et al. [46] conducted systematic scoping review on the nutrition and health benefits of pectin and observed that pectin reduced postprandial blood glucose and insulin response, and increased satiety in healthy and unhealthy participants. According to Ma et al. [7], polysaccharides derived from plants are able to lower postprandial blood glucose by slowing the digestion and absorption of carbohydrates and regulate blood lipids by modulating the activities of key lipid enzymes and cholesterol homeostasis.

The hypothesised mechanisms through which dietary polysaccharides influence glycaemia, insulin levels, lipid metabolism, energy intake and satiety have been widely discussed in the literature, although there appears to be a lack of unanimity amongst researchers regarding the most feasible pathway. These proposed mechanisms include reduction in starch digestion, modulation of gut microbiota, improvement in insulin sensitivity, the preservation of pancreatic β-cells, increasing anti-inflammatory activity and alteration in lipid metabolism [1,5].

The modulation of gut microbiota composition and enhancement of short-chain fatty acid production appear to represent central mechanisms through which dietary polysaccharides influence metabolic homeostasis [47,48]. There is evidence that the intake of poor diets promotes the growth of unhealthy bacteria in the gut and the suppression of beneficial microbes, which could lead to gut microbiota dysbiosis [1,2,3,6]. This disruption of the microbial ecosystem is usually common in metabolic diseases such as T2D and dyslipidaemia [2,3,4]. In particular, people with T2D have been shown to exhibit a notable decrease in butyrate-producing species in their gut microflora [4]. Dysbiosis can lead to the production of metabolites such as lipopolysaccharides and increase in intestinal permeability, which may allow these unfavourable metabolites to translocate into the bloodstream, increasing inflammation [18]. Lipopolysaccharides have also been shown to impede insulin signalling and thus decrease insulin sensitivity, which could impact normal glucose and lipid metabolism [1].

Dietary polysaccharides have been reported to ameliorate glucose dysregulation by modulating gut microbiota dysbiosis, promoting eubiosis and generating advantageous metabolites [2,3,4]. The change in microbial composition following intake of dietary polysaccharides can lead to a decrease in inflammation and oxidative stress, and reduction in intestinal mucosal damage, and control of symptoms of T2D [4]. For example, RS is a functional dietary component, and it has the potential to significantly influence metabolic regulation and gut microbiota modulation [18]. RS can promote the production of short chain fatty acids, regulate glucose metabolism and enhance satiety [11,18]. Short chain fatty acids can induce PI3K phosphorylation and regulate glucose transport, synthesis and catabolism [4]. Byproducts such as butyrate, which are derived from RS, can stimulate glucagon-like peptide-1 (GLP-1) secretion in the gut, enhance insulin secretion, slow gastric emptying, promote insulin sensitivity, and reduce energy intake [18]. As a dietary fibre, RS has been shown to enhance satiety [18].

### 4.1. The Effect of Resistant Starch in Glucose Control and Lipid Metabolism

RS is more resistant to digestion than fully digestible starch resulting in a slower glucose release into the bloodstream that could promote a stable rise in blood sugar levels and enhance satiety [18]. Foods that have a low glycaemic index are digested slowly and do not cause significant fluctuations in postprandial glycemia [8]. Therefore, food with a low GI may have beneficial effect in the prevention and management of diabetes, obesity, and cardiovascular disease [8]. In a systematic review and meta-analysis conducted by Ojo et al. [12], diets with a low glycaemic index were found to be more effective in regulating glycated haemoglobin and fasting blood glucose in patients with T2D compared to the control.

Due to the indigestible nature of RS, it is often considered a functional fibre with beneficial physiological effects, including hypoglycaemic, hypocholesterolaemia and body weight lowering in human subjects [34]. The hypothesised mechanism of RS appears to be increase in production of short chain fatty acid, decreased food intake and promoting satiation and/or satiety [29,34,49]. Increased short chain fatty acid production and exposure to the liver has been linked to increased insulin clearance and increased ghrelin production [29]. Furthermore, reduction in body weight is associated with an increase in insulin sensitivity [34]. RS has also been shown to induce insulin sensitisation in non-insulin-resistant subjects by changing both adipose tissue and skeletal muscle metabolism, including reducing plasma non esterified fatty acid concentration [49].

We examined four studies [27,28,37,38] with different intervention doses, intervention duration and participants’ metabolic status in relation to the effect of RS on glycaemic and lipid parameters. We observed that two of these studies [37,38] with longer durations of 21–28 days significantly reduced glycaemic [37,38] and lipid [38] parameters. On the other hand, one study with shorter duration [27] significantly lowered postprandial free fatty acid concentrations compared to the control. A fourth study [28] with shorter duration reported no significant effect of RS on glucose control in overweight/obese males.

Two of the studies [25,41] with different intervention doses, intervention duration and participants’ metabolic status that reported on the effect of RS4 on glycaemic control and lipid profile found some glycaemic parameters were significantly reduced. A third study [40] with shorter duration reported no significant differences between treatment and control with respect to venous glucose. One study [41] that measured lipid parameters found serum concentrations of lipids were unmodified.

With respect to the effects of intervention doses and metabolic state of participants in studies involving RS2, the Ble-Castillo et al. study [34], had 24 g/day RS2 in obese patients with type 2 diabetes, while Bodinham et al. [29] study had 48 g/day RS2 in healthy young men. Peterson et al. [26] study had 45 g/day RS2 in overweight/obese adults with prediabetes. The findings of the three studies [26,29,34] revealed that irrespective of the intervention dose and the metabolic state of the participants, RS2 did not have significant (*p* > 0.05) effect on glycaemic and lipid parameters compared to some studies with RS4 that had significant effect on glycaemic control. Only one study [36] showed that RS2 decreased responses for glucose.

### 4.2. The Effect of Non-Starch Polysaccharides in Glucose Control and Lipid Metabolism

NSPs also contribute to increased viscosity and could reduce or delay the absorption of carbohydrates and fat in the upper part of the small intestine, which could result in lower blood concentrations of glucose, insulin and cholesterol [8,50]. For example, konjac dietary fibre is a very beneficial soluble dietary fibre, and it is extracted from Amorphophallus konjac, which is gaining prominence as a functional dietary component with benefits for glycaemic regulation [51].

The high viscosity and gel-forming capacity of glucomannan slows gastric emptying and intestinal transit, which delays carbohydrate digestion and absorption and thereby lowers the rate of glucose entry into the bloodstream [52,53,54]. These actions reduce postprandial glucose spikes. In addition, konjac glucomannan is also believed to stimulate satiety hormones (GLP-1, Peptide YY-PYY), enhancing insulin sensitivity and reducing energy intake [53,55]. Konjac glucomannan functions as a fermentable, water-soluble dietary fibre, which increases satiety, regulates metabolism, and delays digestion, making it highly effective in diabetes management, weight control, and the prevention of metabolic syndrome [51,52].

Konjac glucomannan may also control blood glucose through indirect microbiota-mediated pathways [18,40]. It has been suggested that konjac glucomannan as a highly fermentable polysaccharide in the colon, is metabolised by the gut microbes into short-chain fatty acids such as acetate, propionate, and butyrate, which have implications for glucose and lipid metabolism [52,53,56]. In addition, konjac glucomannan can improve satiety by providing bulk and increasing digestion time, thus slowing postprandial glucose uptake, thereby lowering blood glucose and insulin levels [57].

High concentrations of soluble polysaccharides such as β-glucans, alginate, flaxseed gum and guar gum are able to form highly viscous solutions when mixed with fluid and can reduce plasma glucose concentrations [58,59]. The possible mechanism of the action of soluble polysaccharides on plasma glucose may include the slowing of gastric emptying by forming a gel matrix as a result of their water holding capacity, and modulation of digestive processes by decreasing diffusion of nutrients for absorption [58]. Furthermore, there is a decrease in the transport of glucose to absorptive surfaces as a result of alterations in the resistance of digesta to contractile movements within the gastrointestinal tract due to effect of viscous fibres [58]. Ingestion of soluble polysaccharides such as gums may alter blood lipids by decreasing the rate of diffusion, leading to reduction in cholesterol absorption; interfering with bile acid metabolism; reducing emulsification of dietary lipids; and altering short chain fatty acid production [58].

This review also revealed that some studies found that dietary polysaccharides either had neutral or no significant effect on glucose control and/or lipid metabolism. One possible explanation for this is that dietary variability or differences in the composition of the diet among individuals may influence the beneficial effect of dietary polysaccharides [26]. Furthermore, dietary variability resulting from lack of adherence to dietary recommendations during treatment may alter the effect of RS [33]. The differences in the type and amount of RS utilised in these studies, and variations in the participants involved, could lead to differences in outcomes and may further explain the findings of this review with respect to neutral or no significant effect.

Examination of the effect of different intervention doses and intervention duration in relation to konjac glucomannan and konjac products in patients with T2D found that three [39,43,44] of the four studies demonstrated significantly reduced glycaemic parameters compared to the control despite differences in these potential sources of heterogeneity. With respect to lipid profile, only one of these studies [44] showed that mean low-density-lipoprotein-cholesterol concentration after receiving glucomannan was significantly less than that of the placebo. On the other hand, Huang et al. [43] noted no significant effect on lipids, except in triglyceride in patients with hypertriglyceridemia. Vuksan et al. [32] reported that konjac glucomannan had no significant effect on glucose, total, LDL, and HDL cholesterol and triglyceride compared to the control.

The risk of bias assessments has been completed to support the reader in understanding the studies included within this narrative review, and the results of the assessments have shown that the overall strength of the evidence is strong. For example, with respect to the studies showing significant effect of RS on glycaemia, one study [25] of the six studies had low risk of bias in all the domains assessed. Another study [42] in this category had low risk of bias in six domains and unclear risk of bias in one domain. While Park et al. [38] had five low and two unclear risks of bias, Sanders et al. [27] had five low, one unclear and one high, and Kwak et al. [37] had four low and three unclear risks of bias. Mesa Garcia [41] showed low or moderate risk of bias in all the domains, except in relation to bias due to confounding factors, which showed a high risk of bias.

Similarly, of the five studies that showed significant effect of NSP on glycaemia, two studies [44,45] each had a low risk of bias in five domains and an unclear risk of bias in two domains. While the Onyechi et al. study [30] had either low or moderate risk of bias in all the domains, the Ueno et al. [39] and Huang et al. [43] studies showed high risk of bias only in relation to confounding factors.

Of the studies showing that RS had significant effects on insulin, two [29,42] each had a low risk of bias in six of the domains and unclear risk of bias only in one domain. A third study [37] in this category had a low risk of bias in four domains and an unclear risk of bias in three domains. The remaining two studies [35,36] each had a low risk of bias in four domains, an unclear risk of bias in two domains and high risk of bias in one domain.

With respect to NSP and insulin, the study [30] that reported significant reduction in insulin at various postprandial time points had a low or moderate risk of bias in all the domains.

Of the three studies [27,38,44] that found dietary polysaccharides significantly reduced lipid parameters, two [38,44] had a low risk of bias in five domains and an unclear risk of bias in two domains. The third study [27] had a low risk of bias in five domains, an unclear in one domain and a high risk of bias in one domain.

## 5. Limitation of the Review

This review has some limitations that should be acknowledged. The included studies varied considerably in design, sample size, intervention duration and outcome measures, which may limit direct comparisons between studies. Furthermore, the differences in the populations studied and in the types of interventions may have significant effect on heterogeneity and make quantitative synthesis, including meta-analysis difficult. As this was a narrative review, quantitative pooling of data was not performed. Consequently, the findings should be interpreted as a narrative synthesis of the available evidence rather than definitive proof of clinical effectiveness.

## 6. Conclusions

The findings of this review suggest that dietary polysaccharides may contribute to improvements in glucose control and lipid metabolism, although the magnitude and consistency of these effects vary across populations, intervention types and study designs. The most frequently reported beneficial findings related to blood glucose parameters, although substantial heterogeneity remained across studies. Further well-designed studies, including randomised controlled trials with longer durations, are needed to fully establish the role of dietary polysaccharides in the control of blood glucose and lipid parameters.

## Figures and Tables

**Table 1:** An overview of the included studies.

Citation/Country of Study & Year	Type of Study	Aim	Type of Interventions	Results/Findings
Al-Mana and Robertson [28] UK	Randomised, single-blind, crossover clinical trial	To investigate the short-term effects of 48 g RS on appetite, satiety, food intake, and postprandial metabolic responses in overweight/obese males.	Participants consumed 48 g RS incorporated into breakfast and lunch meals versus a placebo (digestible starch), with postprandial measurements taken over 7 h and energy intake assessed at an ad libitum dinner and over 24 h.	RS significantly reduced energy intake at the subsequent ad libitum dinner (*p* = 0.017), but there was no significant reduction in total 24-h energy intake or subjective appetite ratings. RS did not significantly affect insulin or GLP-1 responses.
Arias-Córdova et al. [33] Mexico	Randomised, single-blind, crossover clinical trial using continuous glucose monitoring (CGM)	To evaluate the effects of resistant starch from two sources (native banana starch and high-amylose maize starch) on glycaemic control and variability in patients with T2D when matched for digestible starch.	Participants consumed resistant starch (40 g/day) from either NBS or HMS, compared to the DMS (control), across three 4-day intervention periods with washout phases. Continuous glucose monitoring was used to assess glycaemic outcomes.	Overall, resistant starch showed no consistent benefit on glycaemic regulation in this population.
Arshad et al. [45] Pakistan	Randomised, single-blind, repeated-measures crossover clinical trial	To evaluate the effects of different dietary polysaccharides added to milk on postprandial glycaemic response, appetite, and subsequent food intake in healthy young females.	Participants consumed 250 mL milk (control) or milk enriched with 5 g of carrageenan, guar gum, or alginate. Postprandial glucose and appetite were measured over 120 min, followed by an ad libitum pizza meal to assess energy intake.	Guar gum and alginate significantly reduced postprandial blood glucose and suppressed appetite compared to the control (*p* < 0.0001) and reduced energy intake at the next meal. Guar gum had the strongest effect on satiety and appetite suppression. Carrageenan had minimal impact. Effects were attributed to increased viscosity and delayed gastric emptying.
Au et al. [31] Canada	Randomised, double-blind, crossover postprandial clinical trial	To examine how soy-soluble polysaccharides and flaxseed gum, at varying concentrations and in different food matrices, affect viscosity and postprandial glycaemic and insulinemic responses in healthy adult males.	Interventions: (a) glucose solutions (50 g) with soy-soluble polysaccharides, flaxseed gum, or guar gum, all adjusted to have the same viscosity; (b) dairy drinks containing soy-soluble polysaccharides or flaxseed gum; and (c) dairy puddings with fibre, made with either soy-soluble polysaccharides and κ-carrageenan or flaxseed gum. Controls: a 50 g glucose solution without added fibre, a dairy beverage control with no added fibre and a dairy pudding control containing fibre.	The addition of low-viscosity fibres to glucose solutions did not affect postprandial glucose or insulin responses compared to the fibre-free glucose reference. Both control and fibre-fortified dairy products resulted in lower glucose AUC and GI compared to the glucose reference. Among dairy products, increased viscosity, particularly with flaxseed gum, produced modest reductions in glucose AUC, GI, and peak glucose. Viscosity, rather than fibre dose, modestly influenced glycaemic response.
Ble-Castillo et al. [34] Mexico	Randomised crossover design	To evaluate the effects of native banana starch versus soy milk (control) on body weight and insulin sensitivity in obese patients with T2D.	Participants consumed 24 g/day of NBS (RS2) in 240 mL of water for 4 weeks, and 24 g/day of soy milk (control) in 240 mL of water, using a crossover design with two intervention phases.	The NBS group had significant weight loss reductions compared to control group. BMI in the NBS group was significantly reduced compared to the control (*p* < 0.0001). Fasting insulin levels were significantly reduced from baseline, although not significantly compared to the control. No significant changes were observed in fasting glucose or HbA1c. Lipid metabolism remained largely unchanged.
Bodinham et al. [29] United Kingdom	Randomised, single-blind, crossover clinical trial	To investigate whether consuming 48 g of RS type 2 in mixed meals affects energy intake, subjective appetite, and postprandial glucose and insulin in healthy young men.	On two occasions, at least one week apart, each participant consumed a mixed breakfast and lunch containing 48 g RS2 (Hi-Maize 260) or an energy- and carbohydrate-matched placebo (rapidly digestible starch), followed by an ad libitum dinner and a 24-h diet record.	RS significantly reduced energy intake at the ad libitum meal and over 24 h compared to placebo. Postprandial glucose was similar between treatments, but postprandial insulin response was significantly lower following RS consumption (*p* = 0.029). No differences were observed in subjective appetite ratings. The findings indicate that RS intake reduced energy intake and insulin exposure while maintaining normal blood glucose levels.
Chearskul et al. [44] Thailand	Placebo-controlled crossover trial	To evaluate the effects of glucomannan supplement on glycaemic and lipid indicators in patients with T2D.	A crossover trial with a 2-week washout period. Short term: glucomannan (1 g single dose) or placebo (1 g white rice flour) before a 75 g OGTT Long term: 3 g/day glucomannan for 4 weeks before meal vs. placebo.	Pre-prandial glucomannan ingestion reduced blood glucose rise (*p* < 0.05) without significantly affecting insulin levels. Long-term use reduced 120-min glucose AUC (*p* < 0.05) and decreased LDL-C.
Huang et al. [43] China	Clinical intervention trial in people with T2D	To examine whether konjac food (rich in glucomannan dietary fibre) can lower blood glucose levels, improve lipid profiles, reduce weight and diabetic symptoms, and identify any adverse effects in adults with T2D.	Participants consumed a refined konjac meal (RKM) incorporated into daily foods.	The effects on blood glucose included a significant reduction in fasting blood glucose at 30 and 65 days (*p* < 0.01), a significant reduction in post-prandial blood glucose, a stronger effect than fasting values, and a significant reduction of HbA1c by day 65 (*p* < 0.05).
Kwak et al. [37] South Korea	Randomised, double-blind, placebo- controlled trial	To evaluate if a 4-week dietary intake of resistant starch rice improved blood glucose, oxidative stress, and endothelial function in adults with prediabetes or newly diagnosed T2D.	Daily consumption of rice containing 6.51 g resistant starch vs. refined rice (control) for 4 weeks	Resistant starch rice significantly lowered postprandial glucose and insulin (30 min, *p* = 0.010), reduced glucose and insulin AUCs, and maintained lower glucose at 60 and 120 min after baseline adjustment. Fasting insulin and insulin resistance were reduced.
Lin et al. [42] Taiwan	A randomised crossover study	To evaluate the effects of a resistant starch formula (PPB-R-203) on glucose homeostasis, glycaemic control, and safety in both healthy individuals and patients with T2D.	Test meals compared PPB-R-203-based rice/noodles with conventional white rice/noodles, keeping the same macronutrient ratios (55% carb, 20% protein, 25% fat). An acute 3-h postprandial test was done in healthy participants. A 2-day controlled diet with CGM was conducted in adults with T2D.	In healthy individuals, postprandial glucose and insulin levels were significantly lower following PPB-R-203 meals, with reduced incremental AUC for glucose. In patients with type 2 diabetes, mean blood glucose levels were significantly lower with resistant starch diet. Total glucose AUC and hyperglycaemia AUC (˃10 mmol/L) were also significantly reduced. No increase in hypoglycaemia risk or glycaemic variability.
Mah et al. [25] USA	Double-blind, randomised, controlled, crossover clinical trial	To examine if replacing standard corn starch with tapioca-based resistant starch type 4 in a baked breakfast bar reduces postprandial glucose and insulin responses in healthy adults.	RS4 breakfast bar (tapioca-based RS4, 32 g dietary fibre) vs. macronutrient-matched control bar (standard corn starch and 4 g dietary fibre). A single-test meal with a washout period between interventions.	Consumption of the RS4 breakfast bar resulted in a 22% reduction in median glucose iAUC 0–120 min and a 37% reduction in median insulin iAUC 0–120 min compared to the control (*p* < 0.05). No significant differences were observed in glucose or insulin maximum concentration (Cmax) or time to maximum concentration (Tmax) between groups.
Mesa García et al. [41] Spain	Prospective experimental clinical trial (pre–post intervention design)	To evaluate the effects of a fructose-free, resistant starch type IV-enriched enteral formula on glycaemic control and cardiovascular risk biomarkers in elderly patients with T2D.	Participants were fed exclusively, for 6 weeks, with a diabetes-specific enteral formula enriched with RS type IV and high MUFA, with no fructose content. No parallel control, baseline vs. 6-week comparison	Glycaemic control improved significantly, evidenced by a reduction in HbA1c (*p* < 0.05). Lipid metabolism remained stable, indicating no adverse lipid effects.
Onyechi et al. [30] UK	Randomised controlled crossover feeding study	To investigate the effects of African plant foods rich in non-starch polysaccharides (NSP) on postprandial glucose and insulin responses.	Meals supplemented with Detarium senegalense and Cissus rotundifolia (rich in soluble NSP) vs. control meals.	Participants were normoglycemic at baseline. Postprandial glucose was significantly reduced after detarium and cissus meals (*p* < 0.001 and *p* < 0.0005, respectively), with marked reductions in glucose AUC (↓ 38–62%) and insulin AUC (↓ 36–43% for bread meals). Effects were more pronounced with detarium.
Park et al. [38] South Korea	Randomised double-blind controlled trial	To investigate the effects of resistant starch supplements on blood lipid concentrations, glucose control, insulin response, and immune markers in overweight individuals.	A 24 g/day resistant corn starch vs. regular corn starch for 21 days, consumed with regular diet.	Resistant starch significantly reduced total cholesterol and LDL cholesterol (*p* < 0.05) and significantly lowered fasting blood glucose (*p* < 0.05). No significant effect was observed on insulin levels.
Peterson et al. [26] USA	Randomised, double-blind, placebo- controlled trial	To determine if 12-week resistant starch (RS2) supplementation improves cardiometabolic risk factors in adults with prediabetes.	A 45 g/day resistant starch type 2 (high-amylose maize) vs. isocaloric amylopectin control for 12 weeks.	No significant improvement in glycaemic control, insulin sensitivity, lipid profile, or ectopic fat. A small reduction in HbA1c was observed but was not clinically meaningful and driven by control group changes.
Sandberg et al. [35] Sweden	Randomised cross-over controlled trial	To examine the effects of rye-based evening meals on next-day glucose regulation, appetite, gut hormones, and cardiometabolic risk markers.	Rye kernel bread (high fibre, rich in polysaccharides) vs. white wheat bread, consumed as evening meals (single or 3-day exposure).	Rye significantly reduced postprandial glucose (−23%) and insulin response (−13%) the following morning and increased SCFA (acetate, propionate, butyrate), increased satiety hormones (GLP-1, PYY), reduced hunger, and improved subjective appetite.
Sandberg et al. [36] Sweden	Randomised controlled crossover study	To investigate the effects of whole grain rye products, with and without resistant starch (RS2), on glucose tolerance, gut hormones, inflammation, and appetite regulation in a semi-acute (11–14.5 h) timeframe.	Four rye-based evening meals (rye flour bread, rye flour + kernels, with/without RS2) vs. a white wheat bread control. Outcomes were measured the following morning at fasting and repeatedly up to 3.5 h after a standardised breakfast and at 14.5 h after evening test and reference meals.	Rye kernel + RS significantly reduced postprandial glucose (−27%) and insulin (−21%) responses the next morning (*p* < 0.05) and increased PYY levels (*p* = 0.01), reduced fasting free fatty acids (~−17%), and increased breath hydrogen (*p* < 0.001), indicating fermentation. Rye products improved satiety and reduced hunger, although no change in energy intake or IL-6 was observed.
Sanders et al. [27] USA	Pilot randomised cross-over controlled trial	To assess the effect of resistant starch from cooked and chilled potatoes on insulin sensitivity, metabolic markers, and appetite in adults at risk of T2D.	A total of 300 g/day cooked then chilled potatoes (~18 g resistant starch) vs. isocaloric carbohydrate control over 24 h.	No significant improvement in insulin sensitivity was observed. The resistant starch intervention significantly reduced fasting plasma glucose (*p* = 0.043) and postprandial free fatty acids (*p* = 0.039) and increased postprandial breath hydrogen (*p* = 0.037), indicating enhanced colonic fermentation. Subjective fullness ratings were significantly lower during the resistant starch condition (*p* = 0.002) compared to the control.
Tekin et al. [40] Turkey	Randomised crossover study	To evaluate the glycaemic index (GI) of breads enriched with Type IV resistant starch and assess their effects on appetite and appetite-related hormones.	Consumption of white bread vs. bread enriched with 17% and 24% Type IV resistant starch; glucose used as control; postprandial metabolic and appetite responses measured over 120 min.	RS-enriched bread increased iAUC fullness and significantly altered appetite hormones (↑ GLP-1, ↑ PYY, ↓ ghrelin at key time points, *p* < 0.05). However, GI remained in the medium range (~61–65) and RS did not significantly improve GI compared to white bread.
Ueno et al. [39] Japan	Single-arm, prospective, open-label interventional clinical study over 12 weeks	To investigate whether active daily consumption of konjac and konjac-based foods promotes glycaemic control, body weight, metabolic health and appetite-related hormones.	Participants were instructed to consume at least one konjac product daily for 12 weeks, equivalent to ≥100 g konjac/day. Products included: konjac noodles, rice, desserts and prepared konjac meal items.	Consumption of konjac and konjac products daily significantly improved glycaemic control, reducing HbA1c and fasting blood glucose in adults with T2D. Konjac intake also improved beneficial metabolic markers (notably adiponectin) and supported appetite regulation.
Vuksan et al. [32] Canada	Randomized, double-blind, placebo- controlled, cross-over metabolic trial	To evaluate whether KJM fibre improves metabolic control as measured by glycemia, lipidaemia, and blood pressure in high-risk patients with T2D.	Intervention group: metabolically controlled diet enriched with KJM fibre: KJM biscuits containing ~ 15% KJM flour, of which 69% was glucomannan (0.7 g/100 kcal) Matched placebo group: Same diet enriched with wheat bran fibre (wheat bran biscuits). All participants completed two randomized 3-week periods (separated by a 2-week washout).	When compared to the control, KJM significantly reduced serum fructosamine (−5.7%, *p* = 0.007). No significant differences after correction in glucose. No effects on fasting insulin. KJM as a supplement to conventional therapy may improve cardiometabolic risk factors in high-risk people with T2D.

Abbreviations: Area under the curve (AUC); body mass index (BMI); carbohydrate (carb); continuous glucose monitoring (CGM); code for the retrograded high resistant starch cereal formula (PPB-R-203); digestible maize starch (DMS); glucagon-like peptide (GLP-1); glycaemic index (GI); glycated haemoglobin (HbA1c); high-amylose maize starch (HMS); incremental area under the curve (iAUC); konjac mannan (KJM); low-density lipoprotein (LDL); maximum concentration (Cmax); monounsaturated fatty acids (MUFA); native banana starch (NBS); peptide YY (PYY); resistant starch (RS); resistant starch type 2 (RS2); resistant starch type 4 (RS4); rye kernel test bread (RKB); short chain fatty acids (SCFA); time to maximum concentration (Tmax); type 2 diabetes (T2D); and versus (vs.). ↓ (Decrease); ↑ (Increase).

**Table 2:** Classification of dietary polysaccharides in the included studies and their effects.

Citation/ Country of Study & Year	Source of Polysaccharide	Sub-Group	Component(s)	Effect on Glucose	Effect on Insulin	Effect on Lipid	Mean Daily Energy/ Food Intake	Qualitative Feeling of Satiety/ Appetite
Al-Mana and Robertson [28] UK	Plant	Starch	Resistant starch	No significant treatment effect compared to the placebo	No significant effect	Not applicable	No significant effect	No significant differences in qualitative feelings of satiety and the subjective appetite ratings between RS and the placebo
Arias-Córdova et al. [33] Mexico	Plant	Starch	Resistant starch	No improvement in glycaemic control	No differences were found with respect to insulin	No differences were found with respect to triglycerides or cholesterol	No significant differences in total 24 h energy intake	Not applicable
Arshad et al. [45] Pakistan	Plant	Non-starch polysaccharide	Carrageenan, guar gum, and alginate	Alginate and guar gum resulted in significantly lower cumulative blood glucose (0–170 min) compared to the control	Not applicable	Not applicable	Caloric intake following alginate and guar gum was significantly lower; no differences between the control and carrageenan treatments	The post-treatment average appetite was suppressed by alginate and guar gum
Au et al. [31] Canada	Plant	Non-starch polysaccharide	Guar gum, soy-soluble fibre and flaxseed gum	No significant effect on postprandial glucose	No significant effect on insulin	Not applicable	Not applicable	Not applicable
Ble-Castillo et al. [34] Mexico	Plant	Starch	Native banana starch (NBS; resistant starch)	No significant changes were observed in glycemia and HbA1c levels either across treatments or between treatments	No significant difference in fasting insulin concentration between treatment and control; NBS increased insulin sensitivity	No significant effects of NBS intake on fasting triglycerides, cholesterol levels, HDL-cholesterol, LDL-cholesterol and body fat percentage	Not applicable	Not applicable
Bodinham et al. [29] United Kingdom	Plant	Starch	Resistant starch	No significant effect on postprandial glucose compared to the placebo	Significantly lowered postprandial insulin response compared to the placebo	Not applicable	Significantly lowered energy intake compared to the placebo	No significant effect on subjective appetite ratings
Chearskul et al. [44] Thailand	Plant	Non-starch polysaccharide	Glucomannan	Long-term use of glucomannan significantly reduced postprandial glucose compared to the control	No significant effect on insulin levels	Mean LDL-C concentration after receiving glucomannan was significantly less than that of the placebo	No significant difference in daily total energy intake	The appetite scores during treatments with glucomannan and the placebo did not differ
Huang et al. [43] China	Plant	Non-starch polysaccharide (konjac food)	Glucomannan	Fasting glucose, postprandial glucose and glycated haemoglobin were significantly reduced compared to the control	Not applicable	No significant effect on lipids, except in triglyceride in patients with hypertriglyceridemia	Not applicable	Not applicable
Kwak et al. [37] South Korea	Plant	Starch	Resistant starch	Significantly decreased postprandial glucose compared to the control	A significant reduction in fasting serum insulin	Not applicable	Not applicable	Not applicable
Lin et al. [42] Taiwan	Plant	Starch	Resistant starch	Significantly reduced blood glucose in healthy subjects and in patients with T2D compared to the control	Significantly reduced insulin compared to the control in healthy subjects	Not applicable	Not applicable	Not applicable
Mah et al. [25] USA	Plant	Starch	Resistant starch	Significantly reduced postprandial glucose compared to the control	Significantly reduced insulin compared to the control	Not applicable	Not applicable	Not applicable
Mesa García et al. [41] Spain	Plant	Starch	Resistant starch	Glycated haemoglobin significantly decreased compared to baseline; fasting serum glucose remained unchanged	Fasting insulin remained unchanged	Serum concentrations of lipids were unmodified	Not applicable	Not applicable
Onyechi et al. [30] UK	Plant	Non-starch polysaccharide (Detarium senegalense Gmelin and Cissus rotundifolia)	Not applicable	Significantly reduced plasma glucose compared to the control at most postprandial time points	Significantly reduced insulin at various postprandial time points	Not applicable	Not applicable	Not applicable
Park et al. [38] South Korea	Plant	Starch	Resistant starch	Significantly reduced the mean fasting serum glucose concentrations compared to the control	No significant effect on serum insulin	Significant lowering effects of serum total cholesterol and serum LDL- cholesterol compared to the baseline	Not applicable	Not applicable
Peterson et al. [26] USA	Plant	Starch	Resistant starch	No significant improvement in glycaemic control	No effect on insulin secretion	No effect on total, LDL, or HDL cholesterol; triglyceride; and free fatty acids	Not applicable	Not applicable
Sandberg et al. [35] Sweden	Plant	Starch and non-starch polysaccharide (whole grain rye kernel)	Starch and NSP	Blood glucose was significantly reduced compared to the control	Significantly reduced serum insulin compared to the control; no significant increase in insulin sensitivity	No significant effect on free fatty acids and triglycerides	Not applicable	The rye kernel significantly increased the subjective feeling of satiety at fasting and during the course of the entire experimental day compared to the control; the subjective feeling of hunger and desire to eat were significantly reduced after the RKB compared to the control
Sandberg et al. [36] Sweden	Plant	Whole grain rye kernel with resistant starch 2	Resistant starch 2	Compared to the WWB reference evening meal, the evening meal consisting of RFB/RKB + RS decreased responses for glucose	Compared to the WWB reference evening meal, the evening meal consisting of RFB/RKB + RS decreased responses for insulin	Decreased concentration of free fatty acids	No significant differences in energy intake	Rye flour bread significantly increased the feeling of satiety and decreased feelings of hunger
Sanders et al. [27] USA	Plant	Starch	Resistant starch	Significantly lowered fasting plasma glucose compared to the control	Lower fasting insulin but not significant compared to the control	Significantly lowered postprandial free fatty acid concentrations compared to the control	No significant effect compared to the control	Fullness ratings were significantly lower following intake of RS
Tekin et al. [40] Turkey	Plant	Starch	Resistant starch IV	At the 120th min, there were no significant differences between the treatment and control with respect to venous glucose	At the 120th min., there were no significant differences between the treatment and control with respect to venous insulin	Not applicable	Significantly lower energy intake in 24% resistant starch bread	RS increased satiety compared to white bread
Ueno et al. [39] Japan	Plant	Konjac and konjac products (non-starch polysaccharide)	Glucomannan	Glycated haemoglobin and fasting plasma glucose levels significantly decreased compared to the control	Index for insulin secretion significantly increased	There were no significant changes in LDL- or HDL-cholesterol levels, although triglyceride levels tended to decrease	Not applicable	Appetite remained unchanged in two-thirds of participants
Vuksan et al. [32] Canada	Plant	Konjac mannan (non-starch polysaccharide)	Glucomannan	No significant effect on glucose compared to the control	No significant effect on insulin compared to the control	Significantly reduced total:HDL cholesterol ratio compared to the control; effect on total, LDL, and HDL cholesterol and triglyceride was not significant	Not applicable	Not applicable

## Data Availability

No new data were created or analysed in this study. Data sharing is not applicable to this article.

## Supplementary Materials

The following supporting information can be downloaded at: https://www.mdpi.com/article/10.3390/nu18132143/s1, Table S1: The description and characteristics of included studies.
