# Supplementary material for: Dietary Polysaccharides and the Regulation of Blood Glucose and Lipid Parameters—A Narrative Review
Source: Nutrients. 2026 Jul 2;18(13):2143. doi: 10.3390/nu18132143 (PMC13363572; doi:10.3390/nu18132143)
Supplement: Supplementary file 1 [file nutrients-18-02143-s001.zip › nutrients-4380514-supplementary.pdf]

Supplementary Table S1. The description and characteristics of included studies

Citation/Country of Study & Year	Type of Study	Aim	Participants	Sample Size	Mean Age (Years)	Type of Interventions	Results/Findings
Al-Mana and Robertson [28]<br><br>UK	Randomised, single-blind, crossover clinical trial	To investigate the short-term effects of 48g RS on appetite, satiety, food intake, and postprandial metabolic responses in overweight/obese males.	Healthy overweight/obese males (BMI 28–37 kg/m²), no metabolic or gastrointestinal disease	10 participants<br><br>All male	22 ± 3.7	Participants consumed 48 g RS incorporated into breakfast and lunch meals versus a placebo (digestible starch), with postprandial measurements taken over 7 hours and energy intake assessed at an ad libitum dinner and over 24 hours.	RS significantly reduced energy intake at the subsequent ad libitum dinner ($p = 0.017$), but there was no significant reduction in total 24-hour energy intake or subjective appetite ratings.<br><br>RS did not significantly affect insulin or GLP-1 responses.
Arias-Córdova et al. [33]<br><br>Mexico	Randomised, single-blind, crossover clinical trial using continuous glucose monitoring (CGM)	To evaluate the effects of resistant starch from two sources (native banana starch and high-amylose maize starch) on glycaemic control and glycaemic variability in patients with T2D when matched for digestible starch content.	Adults with T2D (both genders), BMI ≥ 25 kg/m², receiving standard treatment (e.g. metformin, sulfonylureas), with uncontrolled glycaemia (HbA1c > 6.5%).	17 participants (10 with complete CGM data for analysis)<br><br>Male (n=5)<br>Female (n=5)	48.5 ± 9.12	Participants consumed resistant starch (40 g/day) from either NBS or HMS, compared with DMS (control), across three 4-day intervention periods with washout phases.<br><br>Continuous glucose monitoring was used to assess glycaemic outcomes.	Overall, resistant starch showed no consistent benefit on glycaemic regulation in this population.
Arshad et al. [45]<br><br>Pakistan	Randomised, single-blind, repeated-measures crossover clinical trial	To evaluate the effects of different dietary polysaccharides added to milk on postprandial glycaemic response,	Healthy young females (18–30 years)<br><br>Normal BMI, (19.5–25kg/m²)	30 participants	Not explicitly stated (range 18–30 years)	Participants consumed 250 ml milk (control) or milk enriched with 5g of carrageenan, guar gum, or alginate.	Guar gum and alginate significantly reduced postprandial blood glucose and suppressed appetite compared to control (p <

		appetite, and subsequent food intake in healthy young females.	No metabolic disease or dietary restrictions.			Postprandial glucose and appetite were measured over 120 minutes, followed by an ad libitum pizza meal to assess energy intake.	0.0001). Both also reduced energy intake at the subsequent meal.<br><br>Guar gum demonstrated the strongest effect on satiety and appetite suppression. Carrageenan showed minimal impact. Effects were attributed to increased viscosity and delayed gastric emptying.
Au et al. [31]<br><br>Canada	Randomised, double-blind, crossover postprandial clinical trial	To examine how soy-soluble polysaccharides and flaxseed gum, at varying concentrations and in different food matrices, affect viscosity and postprandial glycemic and insulinemic responses in healthy adult males.	Healthy adult males aged 19 to 40 years, with a BMI between 18.5 and 26.0 kg/m², normal fasting glucose and glucose tolerance, and not insulin resistant.	12 participants (11 completed all 11 visits and included in final analysis)	25.3 ± 5.3	Interventions (a) glucose solutions (50g) with 6% soy-soluble polysaccharides, 0.7% flaxseed gum, or 0.23% guar gum, all adjusted to have the same viscosity;<br>(b) dairy drinks containing 1% soy-soluble polysaccharides or 1% flaxseed gum; and<br>(c) dairy puddings with 1% total fibre, made with either soy-soluble polysaccharides and κ-carrageenan or flaxseed gum. in a crossover design.<br><br>Controls: a 50g glucose solution without added fibre, (tested twice), a dairy beverage	The addition of low-viscosity fibres to glucose solutions did not affect postprandial glucose or insulin responses compared to the fibre-free glucose reference.<br><br>Both control and fibre-fortified dairy products resulted in lower glucose AUC and GI compared to the glucose reference.<br><br>Among dairy products, increased viscosity, particularly with flaxseed gum, produced modest reductions in glucose AUC, GI, and peak glucose.<br><br>Conclusion: This study suggests that

						control with no added fibre and a dairy pudding control containing 0.1% total fibre.	viscosity, rather than fibre dose, modestly influenced glycemic response.
Ble-Castillo et al. [34]<br><br>Mexico	Randomised crossover design	To evaluate the effects of native banana starch versus soy milk (control) on body weight and insulin sensitivity in obese patients with T2D.	Obese adults with T2D. Male (n=4) Female (n=24)<br><br>BMI > 30, aged 40–60 years, under medical management but with poor glycaemic control.	30 recruited;<br><br>28 completed the study	51.7 ± 5.6	Participants consumed 24g/day of NBS (RS2) in 240 ml of water for 4 weeks, and 24g/day of soy milk (control) in 240 ml of water, using a crossover design with two intervention phases.	NBS group had significant weight loss reductions compared to control group after 4 weeks of treatment. NBS group lost 1.568kg while control lost 0.3kg ($p = 0.002$) and BMI in the NBS group was significantly reduced compared with control ($p < 0.0001$).<br><br>Fasting insulin levels were significantly reduced from baseline, and insulin sensitivity (HOMA) improved, although not significantly compared to control.<br><br>No significant changes were observed in fasting glucose or HbA1c.<br><br>Lipid metabolism remained largely unchanged.<br><br>Conclusion: NBS functions as a fibre and may contribute to modest

							weight loss and improved insulin sensitivity in obese individuals with type 2 diabetes.
Bodinham et al. [29]<br><br>United Kingdom	Randomised, single-blind, crossover clinical trial	To investigate whether consuming 48g of RS type 2 in mixed meals affects energy intake, subjective appetite, and postprandial glucose and insulin in healthy young men.	Healthy young adult males (aged 19–31 years) with normal BMI and no metabolic or gastrointestinal disorders.	20 participants	25.8 ± 3.7 (SD)	On two occasions, participants attended the investigation unit, at least one week apart.<br><br>Each participant consumed a mixed breakfast and lunch containing 48g RS2 (Hi-Maize 260) or an energy- and carbohydrate-matched placebo (rapidly digestible starch). This was followed by an ad libitum dinner and a 24-hour diet record in a crossover design.	RS significantly reduced energy intake at both the ad libitum meal (5241 vs 5606 kJ, p=0.033) and over 24 hours (12,603 vs 13,949 kJ, p=0.044) compared to placebo.<br><br>Postprandial glucose was similar between treatments, but postprandial insulin response was significantly lower following RS consumption (p = 0.029).<br><br>No differences were observed in subjective appetite ratings. Conclusion: These findings indicate that acute RS intake may reduce energy intake and insulin exposure while maintaining normal blood glucose levels in healthy men.
Chearskul et al. [44]<br><br>Thailand	Placebo-controlled crossover trial	To evaluate the effects of glucomannan supplement on glycemic and lipid indicators in patients with T2D.	People with T2D	20 participants<br><br>Men (n=10)<br>Women (n=10)	Men: 51.40± 2.26<br><br>Women: 51.00± 2.16<br><br>mean±SEM	Short term: Glucomannan (1g single dose) or placebo (1g white rice flour) before a 75g OGTT<br>Long term: 3g/day glucomannan	Pre-prandial glucomannan ingestion reduced blood glucose rise (p<0.05) without significantly affecting insulin levels.

						for 4 weeks before meal vs placebo.<br><br>A crossover trial was performed after 2 weeks washout period.	Long-term use reduced 120-min glucose AUC (p<0.05), decreased LDL-C.
Huang et al. [43] China	Clinical intervention trial in people with T2D	To examine whether konjac food (rich in glucomannan dietary fibre) can lower blood glucose levels, improve lipid profiles, reduce weight and diabetic symptoms, and to investigate any adverse effects in adults with T2D**.**	People with T2D	72 adult subjects with T2D.	Participants were aged 39 to 76 years. Average age: 55 years.	Participants consumed Refined Konjac Meal (RKM) incorporated into daily foods.	Effects on Blood Glucose:<br>• Significant reduction in fasting blood glucose at 30 and 65 days (P<0.01)<br>• Significant reduction in post-prandial blood glucose, stronger effect than fasting values<br>• HbA1c significantly reduced by day 65 (P<0.05)<br>• Largest glucose-lowering effect seen in patients with higher baseline glucose levels
Kwak et al. [37]<br><br>South Korea	Randomised, double-blind, placebo-controlled trial	To evaluate whether 4-week dietary intake of resistant starch rice improves blood glucose, oxidative stress, and endothelial function in individuals with prediabetes or newly diagnosed T2D	Adults with impaired fasting glucose (IFG), impaired glucose tolerance (IGT), or newly diagnosed T2D	90 recruited; 85 completed; adults with impaired fasting glucose, impaired glucose tolerance, or newly diagnosed T2D	~49–52 years	Daily consumption of rice containing 6.51g resistant starch vs refined rice (control) for 4 weeks	Resistant starch rice significantly lowered postprandial glucose and insulin (30 min, P = 0.010), reduced glucose and insulin AUCs, and maintained lower glucose at 60 and 120 min after baseline adjustment. Fasting insulin and insulin resistance were reduced.
Lin et al. [42]<br><br>Taiwan	A randomised	To evaluate the effects of a resistant starch	Three groups – all female	n = 92<br>Group 1 (n=8)	Group 1 – 25.5± 4.0	Test meals compared PPB-R-203-based	In healthy individuals, postprandial glucose and

	crossover study.	formula (PPB-R-203) on glucose homeostasis, glycaemic control, and safety in both healthy individuals and patients with T2D	**Group 1 -** Healthy adults for glycaemic index testing (n=8), **Group 2 -** Healthy adults for glucose and insulin response testing (n=40), **Group 3 -** Adults with T2D diagnosed for >1 year (n=44).	Group 2 (n=40), Group 3 (n=44)	Group 2 - 23.1 ± 2.8 Group 3 - 52.1 ±10.5	rice/noodles with conventional white rice/noodles, keeping the same macronutrient ratios (55% carb, 20% protein, 25% fat).<br><br>An acute 3-hour postprandial test was done in healthy participants.<br><br>A 2-day controlled diet with CGM was conducted in type 2 diabetes.	insulin levels were significantly lower following PPB-R-203 meals, with reduced incremental AUC for glucose.<br><br>In patients with type 2 diabetes, mean blood glucose levels were significantly lower with the resistant starch diet (7.4 vs 7.9 mmol/L, P=0.023).<br><br>Total glucose AUC and hyperglycaemia AUC (> 10mmo/L) were also significantly reduced.<br><br>There was no increase in hypoglycaemia risk, or glycemic variability indices<br><br>Conclusion: This finding suggests that the RS formula can be used to safely reduce postprandial hyperglycemia in individuals with T2D.
Mah et al. [25]<br><br>USA	Double-blind, randomised, controlled, crossover clinical trial	To examine whether replacing standard corn starch with tapioca-based resistant starch type 4 in a baked breakfast bar	Healthy adults. (male: n = 10) and (female: n=11, non-pregnant and non-lactating)	n=21	34.14 ± 8.62 years.	RS4 breakfast bar (tapioca-based RS4, 32 g dietary fibre) vs macronutrient-matched control bar (standard corn	Consumption of the RS4 breakfast bar resulted in a 22% reduction in median glucose iAUC 0-120 min and a 37% reduction in median

		reduces postprandial glucose and insulin responses in healthy adults	BMI between 18.5 and 26.9 kg/m² and non-glycaemic (fasting blood glucose ≤110 mg/dL).			starch and 4 g dietary fibre).<br><br>A single test meal crossover study with a washout period between interventions.	insulin iAUC 0-120 min compared to control (P< 0.05)<br><br>No significant differences were observed in glucose or insulin maximum concentration (Cmax) or time to maximum concentration (Tmax), between groups.<br><br>Conclusion: A practical reduction in available carbohydrate results in improved acute postprandial glycemic and insulinemic responses in healthy adults.
Mesa García et al. [41]<br><br>Spain	Prospective experimental clinical trial (pre–post intervention design)	To evaluate the effects of a fructose-free, resistant starch type IV-enriched enteral formula on glycaemic control and cardiovascular risk biomarkers in elderly patients with T2D.	Elderly patients (male, n=9) (female, = 32) with T2D requiring enteral nutrition	n= 41 participants (elderly T2D patients).	78.9 ± 2.8 (SEM)<br><br>78.9 ± 17.9 (SD)	Participants were fed exclusively for 6 weeks with a diabetes-specific enteral formula enriched with RS type IV and high MUFA, with no fructose content.<br><br>No parallel control, baseline vs 6week comparison	Glycaemic control improved significantly, evidenced by a reduction in HbA1c (6.1% to 5.8%, $p < 0.05$). Lipid metabolism remained stable, indicating no adverse lipid effects.<br><br>Conclusion: Overall, the intervention improved glycaemic control and reduced cardiovascular risk in elderly patients with diabetes without negatively affecting lipid profiles.

Onyechi et al. [30]<br><br>UK	Intervention crossover feeding study	To investigate the effects of African plant foods rich in non-starch polysaccharides (NSP) on postprandial glucose and insulin responses	Healthy adults (non-diabetic)	n = 5 (stew meals), n = 10 (bread meals)	Stew group: 30.8 ± 2.4 years; Bread group: 28.7 ± 2.3 years	Meals supplemented with Detarium senegalense and Cissus rotundifolia (rich in soluble NSP) vs control meals	Participants were normoglycaemia at baseline (fasting plasma glucose 4.1 ± 0.2 mmol/L; range 3.5–4.8 mmol/L). Postprandial glucose was significantly reduced after detarium and cissus meals ($P < 0.001$ and $P < 0.0005$, respectively), with marked reductions in glucose AUC (↓ 38–62%) and insulin AUC (↓ 36–43% for bread meals). Effects were more pronounced with detarium. The glucose-lowering effect is attributed to soluble NSP, likely increasing intestinal viscosity and reducing carbohydrate digestion and absorption.
Park et al. [38]<br><br>South Korea	Randomised double-blind controlled trial	To investigate the effects of resistant starch supplementation on blood lipid concentrations, glucose control, insulin response, and immune markers in overweight individuals.	Overweight/obese adults (>120% ideal body weight), female participants recruited from Seoul	n = 25 (Control n=13; Intervention n=12)	~43 years (range 26–57 years)	24g/day resistant corn starch vs regular corn starch for 21 days, consumed with regular diet.	Resistant starch significantly reduced total cholesterol and LDL cholesterol ($p<0.05$), and significantly lowered fasting blood glucose ($p<0.05$). No significant effect was observed on insulin levels. Triglycerides remained unchanged in the intervention group but

							increased in controls.
Peterson et al. [26] USA	Randomised, double-blind, placebo-controlled trial	To determine whether 12-week resistant starch (RS2) supplementation improves cardiometabolic risk factors in adults with prediabetes	Overweight/obese adults with prediabetes (BMI ≥27 kg/m², aged 35–75 years)	68 randomised; 59 completed the study	55 ± 10 year	45 g/day resistant starch type 2 (high-amylose maize) vs isocaloric amylopectin control for 12 weeks	No significant improvement in glycaemic control, insulin sensitivity, lipid profile, or ectopic fat. A small reduction in HbA1c was observed but was not clinically meaningful (0.1 ± 0.2% (Δ = −1 ± 2 mmol/mol) and driven by control group changes.
Sandberg et al. [35] Sweden	Randomised cross-over controlled trial	To examine the effects of rye-based evening meals on next-day glucose regulation, appetite, gut hormones, and cardiometabolic risk markers	Healthy young adults (non-smokers, BMI 19–25kg/m², no metabolic disease or food allergies) 9 men and 10 women	n =19	25.6 ± 3.5 years	Rye kernel bread (high fibre, rich in polysaccharides) vs white wheat bread, consumed as evening meals (single or 3-day exposure)	Rye significantly reduced postprandial glucose (−23%) and insulin response (−13%) the following morning. It increased SCFA (acetate, propionate, butyrate), increased satiety hormones (GLP-1, PYY), reduced hunger, and improved subjective appetite.
Sandberg et al. [36] Sweden	Randomised controlled crossover study	To investigate the effects of whole grain rye products, with and without resistant starch (RS2), on glucose tolerance, gut hormones, inflammation, and appetite regulation in a semi-acute (11–14.5h) timeframe	Healthy adults (BMI 19–25 kg/m², non-smokers, no metabolic disease)	21 participants completed the study	25.3 ± 3.9 years	Four rye-based evening meals (rye flour bread, rye flour + kernels, with/without RS2) vs white wheat bread control; Outcomes were measured the following morning at fasting and repeatedly up to 3.5h after a standardised breakfast and at 14.5h after	Rye kernel + RS (RFB/RKB + RS) significantly reduced postprandial glucose (−27%) and insulin (−21%) responses the next morning (P < 0.05). It increased PYY levels (P = 0.01), reduced fasting free fatty acids (~−17%), and increased breath hydrogen

						evening test and reference meals	(P < 0.001), indicating fermentation. Rye products improved satiety and reduced hunger, although no significant change in energy intake or IL-6 was observed.
Sanders et al. [27]<br><br>USA	Pilot randomised cross-over controlled trial	To assess the effect of resistant starch from cooked and chilled potatoes on insulin sensitivity, metabolic markers, and appetite in adults at risk of T2D.	Overweight/obese adults at risk of T2D (BMI 27–39.9)	n = 19	48.0 ± 2.9 years	300g/day cooked then chilled potatoes (~18g resistant starch) vs isocaloric carbohydrate control over 24 hours	No significant improvement in insulin sensitivity was observed. However, the resistant starch (potato) intervention significantly reduced fasting plasma glucose (P = 0.043) and postprandial free fatty acids (P = 0.039), and increased postprandial breath hydrogen (P = 0.037), indicating enhanced colonic fermentation. A trend toward lower fasting insulin was noted (P = 0.077). Subjective fullness ratings were significantly lower during the resistant starch condition (P = 0.002) compared with control.
Tekin et al. [40]<br><br>Turkey	Intervention crossover study	To evaluate the glycaemic index (GI) of breads enriched with Type IV resistant	Healthy adults (non-smokers, BMI 18.5-24.9, no	10 participants (5 male, 5 female)	31.3 ± 7.95 years	Consumption of white bread vs bread enriched with 17% and 24% Type IV resistant	The iAUC fullness values of RS bread were higher than those of both glucose and white bread.

		starch and assess their effects on appetite and appetite-related hormones	metabolic disease)			starch; glucose used as control; postprandial metabolic and appetite responses measured over 120 minutes	RS-enriched bread increased iAUC fullness and significantly altered appetite hormones (↑GLP-1, ↑PYY, ↓ghrelin at key time points, $p<0.05$). However, GI remained in the medium range (~61-65) and RS did not significantly improve GI compared to white bread.
Ueno et al. [39] Japan	Single-arm, prospective, open-label interventional clinical study over 12 weeks.	To investigate whether active daily consumption of konjac and konjac-based foods promotes glycaemic control, body weight, metabolic health and appetite-related hormones.	Participants with T2D.	26 patients with T2DM.	Participants aged 29–75 years. Mean age ≈ 55.4 years.	Participants were instructed to consume at least one konjac product daily for 12 weeks, equivalent to minimum ≥100g konjac/day. Products included: •Konjac noodles •Konjac rice •Konjac desserts •Various prepared konjac meal items.	Consumption of konjac and konjac products daily, significantly improved glycaemic control, reduced HbA1c and fasting blood glucose in adults with T2D. Konjac intake also improved beneficial metabolic markers (notably adiponectin), supports appetite regulation, may aid weight control, and is safe.
Vuksan et al. [32]<br><br>Canada	Randomized, double-blind, placebo-controlled, cross-over metabolic trial	To evaluate whether KJM fiber improves metabolic control as measured by glycemia, lipidemia, and blood pressure in high-risk patients with T2D.	People with T2D	11 participants<br><br>n=5 men and n= 6 women	Men: 62 ± 8<br><br>Women: 59 ± 7<br><br>Mean ± SD	Intervention group: NCEP step 2 metabolically controlled diet enriched with KJM fibre: KJM biscuits containing ~ 15% KJM flour of which 69% glucomannan (0.7g/100kcal)	When compared with control, KJM significantly reduced serum fructosamine (−5.7%, $p=0.007$). No significant differences after correction in glucose. No effects on fasting insulin.

	Matched placebo group: Same diet enriched with Wheat bran fibre (Wheat bran biscuits).<br><br>All participants completed two randomized 3-week periods (separated by a 2-week washout).	KJM as a supplement to conventional therapy may improve cardiometabolic risk factors in high-risk people with T2D.

Abbreviations: Area under the curve (AUC); Body mass index (BMI); Carbohydrate (carb); Con-tinuous glucose monitoring (CGM); continuous overlapping net glycemic action (CONGA); Code for the retrograded high resistant starch cereal formula (PPB-R-203); Digestible maize starch (DMS); Glucagon-like peptide (GLP-1); Glycaemic index (GI); Glycaemic risk assessment in diabetes equation (GRADE); Glycated haemoglobin (HbA1c); High-amylose maize starch (HMS); High-density lipo-protein (HDL); Homeostasis Model Assessment (HOMA); Homeostatic Model Assessment 2 for Insulin Resistance (HOMA2-IR); Incremental area under the curve (iAUC); Insulinemic index (II); Kilojoules (kJ); Konjac mannan (KJM); Low-density lipoprotein (LDL); Maximum concentration (Cmax); Monocyte chemotactic protein 1 (MCP-1); Monounsaturated fatty acids (MUFA); Native banana starch (NBS); National cholesterol education programme (NCEP); Peptide YY (PYY); Plasminogen activator inhibitor 1 (PAI-1); Resistant starch (RS); Resistant starch type 2 (RS2); Re-sistant starch type 4 (RS4); Rye kernel test bread (RKB); Short chain fatty acids (SCFA); Soluble endothelial selectin (sE selectin); Soluble vascular cell adhesion molecule-1 (sVCAM-1); Standard deviation (SD); Time to maximum concentration (Tmax); Type 2 diabetes (T2D); Versus (vs); White wheat flour bread (WWB); Whole grain rye flour and rye kernels bread (RFB/RKB).
